# 

**Published:** 1907-01-12

**Authors:** 


### BOOKS RECEIVED.

A. AND C. BLACK.

"The Englishwoman's Year-book," 1907-8.
"The Writers' and Artists' Year-book," 1907.

J. B. LIPPINCOTT CO.

"International Clinics," vols. ii. and iii. Sixteenth Series.

MACMILLAN AND CO.

"The Health Reader."—I. By C. E. Shelly, M.D., and E. Stenhouse, B.Sc.

SMITH, ELDER AND CO.

"Climatotherapy and Balneotherapy." By Sir Hermann Weber, M.D., and F. Parkes Weber, M.D.

WARD, LOCK AND CO.

"Mrs. Beeton's Book of Household Management." (New edition).

### NOTICE TO CORRESPONDENTS.

All MSS., Letters, Books for Review, and other matters intended for the Editor should be addressed THE EDITOR, "THE HOSPITAL," THE HOSPITAL BUILDINGS, 28 & 29 SOUTHAMPTON STREET, STRAND, W.C.

The Editor cannot undertake to return rejected MS., even when accompanied by stamped directed envelope.

### Notice to Advertisers and Subscribers.

All Advertisements, Orders for Copies of THE HOSPITAL, and Business Communications should be addressed to **THE MANAGER (Not to the Editor).** THE HOSPITAL, 28 & 29 Southampton Street, Strand London, W.C.

The Cost of Subscription, post free, is as follows:—Per week, 3½d.; per quarter, 3s. 3d.; per half-year, 6s. 6d.; per annum, 13s. Foreign and Colonial:—Per annum, 19s. 6d., payable, in all cases, in advance.

**NOTICE.—Vol. XL. of "The Hospital" (April to September 1906), in handsome cloth binding will shortly be on sale at the office of this Journal price 10s. 6d. Cases for binding the Numbers contained in this Volume 2s. Index to Vol. XL., 3½d., post free.**

**The Monthly Part for January is now ready. Price 1s., by post, 1s. 3d.**

